# Posner-Schlossman syndrome relapse following inactivated COVID-19 vaccination in China

**DOI:** 10.3389/fpubh.2022.1051378

**Published:** 2023-01-13

**Authors:** Qilian Sheng, Yanan Sun, Ruyi Zhai, Xintong Fan, Yue Ying, Xiangmei Kong

**Affiliations:** ^1^Eye Institute and Department of Ophthalmology, Eye & ENT Hospital, Fudan University, Shanghai, China; ^2^National Health Commission Key Laboratory of Myopia (Fudan University), Shanghai, China; ^3^Key Laboratory of Myopia, Chinese Academy of Medical Sciences, Shanghai, China; ^4^Shanghai Key Laboratory of Visual Impairment and Restoration, Shanghai, China

**Keywords:** Posner-Schlossman syndrome, COVID-19, vaccine, inactivated, uveitis

## Abstract

**Introduction:**

This retrospective study aims to present the characteristics of Posner-Schlossman syndrome (PSS) relapse following inactivated COVID-19 vaccination.

**Methods:**

From 2020 to 2022, 12 out of 106 PSS patients undergoing relapses after any dose of inactivated COVID-19 vaccines were enrolled. Medical histories, information on the vaccination and systemic adverse events were collected. Patients were treated with corticosteroids, intraocular pressure (IOP)-lowering drugs and systemic immunosuppressive agents (if needed). Daily regimen and release course were noted.

**Results:**

The recurrence rate after vaccination was 11.32% (12/106, 95% CI: 5.29%–17.35%) among 106 PSS patients we surveyed. All the 12 patients were inoculated with inactivated COVID-19 vaccines developed by Sinopharm, China. The mean time of relapse was 5.27 ± 3.72 days (range: 1–13 days, median: 4 days). Higher IOP and more keratic precipitates (KPs) were seen in the relapse following vaccination (33.55 ± 12.99 mmHg, 91.67% had KPs compared to 25.38 ± 3.80 mmHg, 33.33% had KPs in previous relapse, *P* = 0.009). The mean release course was 30.71 ± 34.74 days for the relapse following vaccination and 7.33 ± 6.51 days for previous relapses. The attack frequency before and after vaccination was 3.56 ± 2.07 and 9.11 ± 7.34 times per year (*P* = 0.044). Higher daily doses of corticosteroids, IOP-lowering drugs and ganciclovir were needed to maintain stable course, though the difference did not reach statistical significance.

**Discussion:**

More frequent relapses and harder control of IOP were found in PSS relapse following COVID-19 vaccination. Ophthalmologists need to be aware of the group vulnerability and take precautions, though the pathogenesis is still under investigation.

## 1. Introduction

Posner-Schlossman syndrome (PSS), also known as glaucomatocyclitic crisis, is a recurrent, unilateral uveitis accompanied by elevated intraocular pressure (IOP) and typical keratic precipitates (KPs) on the corneal endothelium (1). PSS is now identified to cause glaucomatous fundus impairment through frequent relapses and IOP peaks, though firstly recognized benign and self-limited (2). Topical IOP-lowering drugs and corticosteroid eye drops are currently used to control relapses and might gradually tapered after the acute attack has been managed. However, disease progression and increased relapse frequency troubled both clinicians and patients. No certain predisposing factor for PSS has been confirmed yet; those being mostly observed include infection, tiredness, staying up late into the night, systemic immunology abnormalities, etc. Since the pathogenesis of PSS has not been clarified, current medication mainly aims at releasing uveitic attacks and avoiding fundus and optic nerve damage caused by elevated IOP (1).

The outbreak of coronavirus disease 2019 (COVID-19), which was caused by the severe acute respiratory syndrome coronavirus 2 (SARS-CoV-2), had posed severe threat to public health maintenance worldwide (3). With the achievement of useful vaccines against COVID-19, the high transmission and mortality rate of COVID-19 could be effectively restricted (4). Multiple platforms of COVID-19 vaccines have been developed based on protein subunit, viral vector, inactivated viruses, mRNA etc. (5). To date, inactivated vaccines from Wuhan Institute of Biological Products (Sinopharm, China) have been widely deployed in China (6). Several ocular adverse events had been reported in small cohorts (7–9), among which inflammatory eye disease, acute uveitis and reactivation of immunological uveitis were noticed (8, 10, 11). The immunological underpinnings of various eye diseases may reflect a derangement in systemic immunity despite the eye being a prototypic immune-privileged organ (12). Apart from viral infection (13), evidence on dysfunctional adaptive immune system was previously found in PSS (14, 15). The immunogenicity of COVID-19 vaccines could potentially induce or trigger autoimmune diseases even without application of systemic immunosuppressive therapy. Our study reported the recurrence after COVID-19 vaccination in several PSS patients and compared it with previous relapses of the same group. Though a causal relationship could not be established, it would be of great importance to remind these patients the risk of vaccination and furthermore, provide possible precautions in advance.

## 2. Materials and methods

### 2.1. Participants

From 2020 to 2022, 106 PSS patients diagnosed and treated in Eye & ENT Hospital of Fudan University, Shanghai, China were surveyed and examined after inactivated COVID-19 vaccination. In total, 12 patients underwent relapse within 15 days after vaccination were enrolled and reviewed in our study. The study protocol was in accordance with the Declaration of Helsinki, and was approved by the Ethics Committee of EENT Hospital of Fudan University; all participants signed informed consent in written form.

The inclusion criteria were as follows: (1) patients aged 10–80, clearly diagnosed PSS: recurrent attacks of mild, unilateral, non-granulomatous anterior uveitis accompanied by markedly elevated IOP, small white KPs on the endothelial surface of the cornea, open angle, no posterior synechia, and no inflammatory lesions in the posterior segment of the eye; the IOP and anterior chamber inflammation returned normal between attacks; the fellow eye was completely normal under current ophthalmic examinations; (2) patients underwent PSS relapses after any dose of inactivated COVID-19 vaccines.

The exclusion criteria were as follows: (1) elevated IOP caused by any other known factor; (2) age-related macular degeneration, proliferative and non-proliferative diabetic retinopathy, or significant risk of glaucomatous vision loss caused by medical washout of IOP-lowering drugs; (3) clinically observable corneal dystrophy, infectious keratitis, and central corneal thickness of < 480 or more than 620 μm; (4) previous ocular trauma, idiopathic uveitis or non-infectious uveitis, or comorbid primary open-angle glaucoma; (5) kidney or liver dysfunction, and during active phase of other systematic diseases; (6) pregnant or lactating women.

### 2.2. Data collection

Demographics and medical histories of previous relapses were reviewed; thorough ophthalmic examinations were performed at the visit of relapse following COVID-19 vaccination, including IOP measurement (Goldmann applanation tonometer, Keeler Co., Ltd., London, UK), best-corrected visual acuity (BCVA, measured in logMAR), anterior chamber manifestations under the slit-lamp and medication regime (commercially available topical drugs including single or complex of α-agonists, β-blockers and carbonic anhydrase inhibitors, corticosteroids, 2% ganciclovir eye drops produced in the Pharmacy of EENT Hospital).

### 2.3. Statistical analyses

Statistics were analyzed using GraphPad Prism Software (2022, San Diego, CA, USA) and SPSS software (IBM Corp., version 21.0, Armonk, NY, USA). Mann–Whitney U test and *t*-test were used to compare differences between IOP, drug dosage and other numerical variables. χ2 and Fisher exact test were used to compare gender and other categorial variables between groups. A *P* < 0.05 was considered statistically significant.

## 3. Results

**Figure 1:**
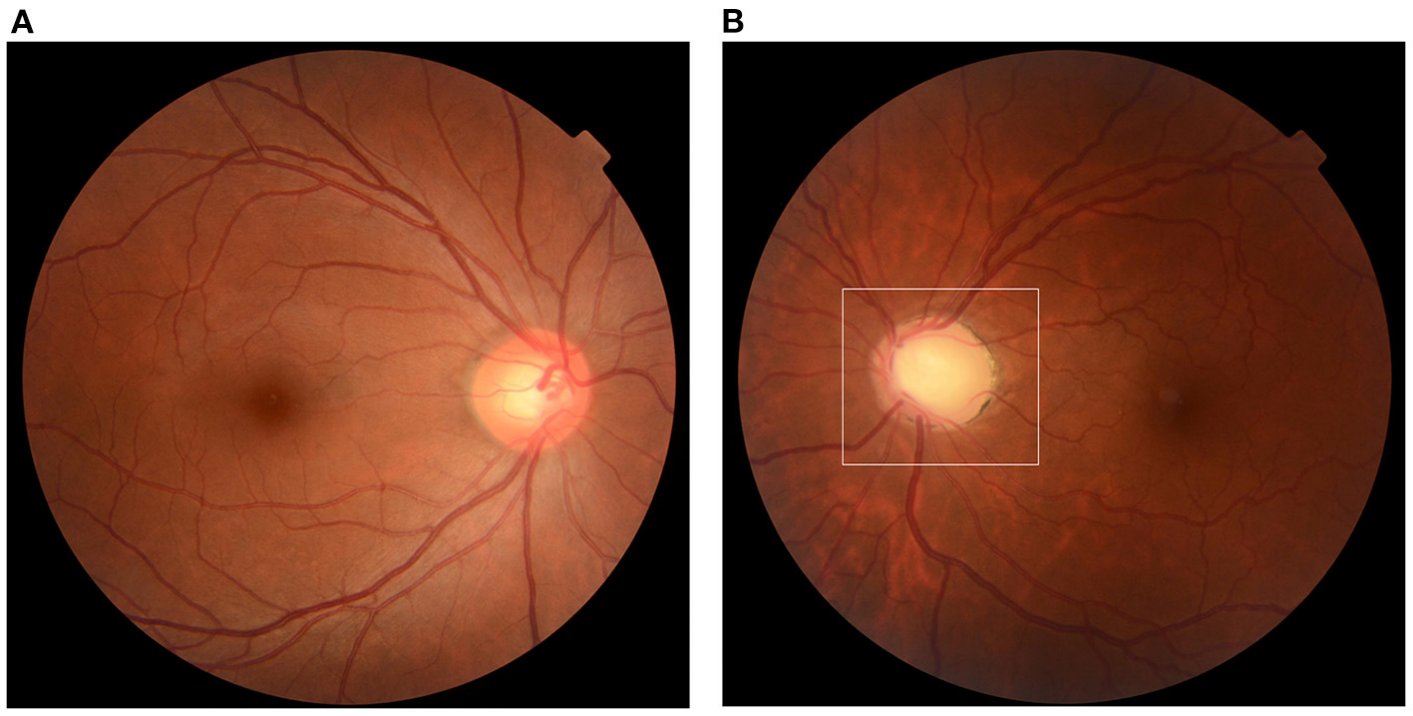
Optic disc cupping. Fundus photograph captured from a 41-year-old female patient. **(A)** Normal fundus of the right eye, CDR = 0.5. **(B)** Significant cupping of the left eye (area in the white frame) compared to the right eye, CDR = 0.8. CDR, cup-to-disc ratio.

The relapse rate following inactivated COVID-19 vaccination was 11.32% (12/106, 95% CI: 5.29–17.35%). The demographics and previous medication of the 12 patients were recorded and presented in Table 1. Among all the 106 patients, 90 reported they never underwent relapse after any vaccine (84.91%, 95% CI: 78.10–91.72%); 3 female patients underwent relapse after inoculation of human papillomavirus (HPV) vaccine (2.83%, 95% CI: 1.22–4.44%); 1 after 4-valent and 2 after 9-valent. One patient underwent relapse after rabies vaccine previously (0.94%). These 4 patients did not report relapse after any dose of inactivated COVID-19 vaccination. None of the 106 patients had caught natural infection of SARS-CoV-2 since the outbreak of COVID-19 (0%). Common reasons reported by all the patients generally included: constant fatigue or staying up late 55.66% (59/106, 95% CI: 46.20–65.12%), no certain causes or unexplained relapses 34.91% (37/106, 95% CI: 25.84–43.98%), emotions, stress or menstrual cycles 2.83% (3/106, 95% CI: −0.33%-5.99%), catching infectious disease (infectious keratoconjunctivitis, influenza) 2.83% (3/106, 95% CI: −0.33–5.99%), alcohol consumption or specific food 1.89% (2/106, 95% CI: −0.70–4.48%), allergic disease attacks (erythra, asthma, rhinitis and other autoimmune diseases, etc.) 1.89% (2/106, 95% CI: −0.70–4.48%). Among the 12 patients, one patient had received the first dose of COVID-19 vaccination till now; 7 had received the first two doses and the other 4 had received all the three doses. The COVID-19 inactivated vaccines were all from Sinopharm Life Sciences Co., Ltd., China and were inoculated according to standardized procedure. All patients were inoculated at the quiescent stage of PSS. None of them had undergone relapse after any other vaccine before. In total, 4 (33.33%) patients underwent relapse after the first dose; 4 (33.33%) after the second, 1 (8.33%) after the third and 2 (16.67%) underwent two relapses after the first and second dose. The mean time for relapse following COVID-19 vaccination was 5.27 ± 3.72 days (range: 1–13 days, median: 4 days). All the patients reported more frequent relapses and harder control of IOP after vaccination; one patient's recurrence could not be controlled without corticosteroids, while his previous relapses were usually released within 1 month under low dosage of topical corticosteroids (2 drops/day). In previous relapses, possible incentives frequently reported were constant fatigue and emotions (75.00%, 8/12), specific food (8.33%, 1/12) and uncertain causes (16.67%, 2/12). Among the rest 94 patients, 89 reported a similar frequency of relapse within 3 months after vaccination (3.54 ± 3.38 times/year), 5 patients were excluded from the frequency analysis due to increased frequency caused by their poor treatment compliance, including self-discontinuation of medication or changing drugs or formulations without prescription, etc. All of the rest 94 patients had inoculated at least one dose of the inactivated COVID-19 vaccine and no relapse had been reported within 3 months after vaccination. Figure 1 showed significant cupping in the left eye captured from one enrolled patient.

**Table 1:** The demographics and previous medication of PSS patients undergoing relapses following COVID-19 vaccination.

**Patients' demographics**	**Value**
Gender (M/F)	7/5
Age (years)	34.30 ± 10.74
Systemic diseases	0/12
Immune system abnormalities	1 diagnosed hyperthyroidism, 11 normal
Systemic medication	0/12
PSS disease course (years)	8.48 ± 6.27
Laterality (OD/OS)	7/5
Ocular medication course (years)	8.40 ± 6.38
Anti-glaucoma surgical history	0/12
Ocular laser surgery	0/12
Aqueous humor viral pathogens IgG test	1 RV+, 2 CMV+, 2 viral pathogens free, 7 no test
BCVA of AE	0.47 ± 0.35
BCVA of FE	0.29 ± 0.32
CED of AE (cells/mm^2^)	2,415.67 ± 453.41
CED of FE (cells/mm^2^)	2,765.67 ± 147.76
CCT of AE (μm)	556.00 ± 5.66
CDR of AE	0.47 ± 0.06
Visual field MD (dB)	−3.65 ±−5.13
RNFL of AE (μm)	71.00 ± 11.31

M, male; F, female; PSS, Posner-Schlossman syndrome; OD, oculus dexter, the right eye; OS, oculus sinister, the left eye; CMV, cytomegalovirus; RV, rubella virus; IOP, intraocular pressure; BCVA, best-corrected visual acuity, measured by logMAR; CED, corneal endothelial cell density; CCT, central corneal thickness; CDR, cup-to-disc ratio; MD, mean deviation; RNFL, retinal nerve fiber layer. AE, the affected eye; FE, the fellow eye.

### 3.1. Severe uveitic attack and hard control in the relapse following vaccination

**Figure 2:**
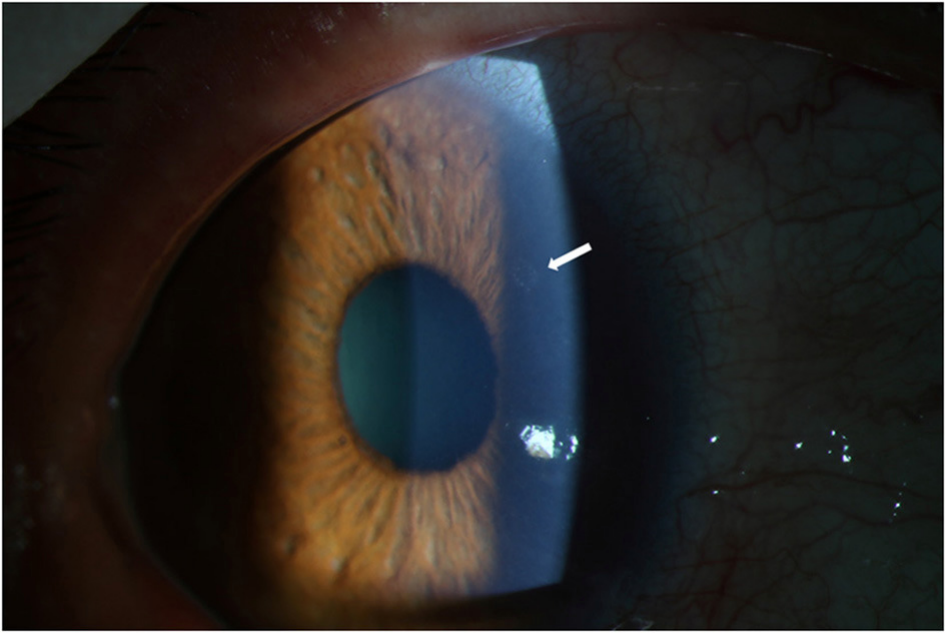
Fresh KPs in the relapse following COVID-19 vaccination. Captured from the right eye of a 47-year-old male patient. Typical KPs (white arrow) appeared on the upper corneal endothelium at the third day after COVID-19 vaccination. KP, keratic precipitate.

The ocular manifestations and ophthalmic examinations of typical previous relapse and the relapse following inactivated COVID-19 vaccination were recorded and presented in Table 2. More patients had fresh KPs in the relapse following COVID-19 vaccination (91.67%) than previous relapses (33.33%, *P* = 0.009, typical fresh KPs were shown in Figure 2). Five patients had more than 10 mutton-fat KPs (diffused on the lower corneal endothelium) and three patients had 1–5 fresh KPs. The IOP of relapse following vaccination was higher than that of previous relapse, though the difference did not reach statistical significance. The dosage of corticosteroids, GCV and IOP-lowering drugs needed to control relapse following vaccination was higher than those of previous relapses. One patient needed oral ganciclovir and corticosteroids to control lasting high IOP; oral immunosuppressive agents were also given by local hospital before coming to our department. In terms of the release course, only three patients controlled the relapse following vaccination within 1 week; three patients controlled relapses in 3 months; the other 6 patients' relapses could not be controlled within 3 months (iterative relapses during continuing medication, even under topical corticosteroids 4 times per day). For these patients, loteprednol etabonate eye drops (Bausch and Lomb Incorporated, USA) and 0.1% fluorometholone (Santen Pharmaceuticals, Japan) were changed to stronger corticosteroid suspensions such as 0.1% dexamethasone (ALCON-COUVREUR, Belgium) and 1% prednisolone (Allergan Pharmaceuticals, Ireland); compound suspensions of anti-glaucoma agents were also used instead of single agent; 0.1% Tacrolimus eye drops were needed additionally in one patient.

**Table 2:** The comparison of previous relapses and relapse following COVID-19 vaccination.

	**Previous relapse**	**Relapse following COVID-19 vaccination**	***P*-value**
IOP of AE (mmHg)	25.38 ± 3.80	33.55 ± 12.99	0.196
IOP of FE (mmHg)	17.98 ± 0.82	19.75 ± 3.23	0.327
Conjunctival congestion	0/12	0/12	/
Ciliary congestion	0/12	0/12	/
Corneal edema	0/12	0/12	/
Fresh KPs	4/12	11/12	**0.009** ^*^
Shallow anterior chamber	0/12	0/12	/
Tyndall effect	1/11	0/12	0.500
Anterior chamber cell	1/11	0/12	0.500
Iris depigmentation	1/11	1/11	/
Corticosteroids dosage (drops/day)	2.67 ± 1.37	3.22 ± 1.39	0.460
GCV dosage (drops/day)	1.33 ± 2.07	1.56 ± 2.01	0.839
IOP-lowering drugs (drops/day)	2.83 ± 1.94	3.11 ± 2.89	0.841
Systemic immunosuppressive medication	0/12	1/11	0.500
Release course (days)	7.33 ± 6.51	30.71 ± 34.74	0.295

IOP, intraocular pressure; AE, the affected eye; FE, the fellow eye. GCV, ganciclovir. Statistically significant values were marked in bold with

^*^.

### 3.2. Higher regimen was needed to maintain current disease condition

After the acute relapse was controlled, the regimens applied to maintain stable intervals were compared in Table 3. Significantly more relapses were seen in patients after vaccination (*P* = 0.044). The daily regimens of corticosteroids, GCV and IOP-lowering drugs were all higher than those before vaccination, though statistical significance was not reached. The increase of daily regimen may bring about ocular adverse events, such as corneal dystrophy and steroid-induced ocular hypertension, which in turn worsened the sustain of high IOP (though this was not observed in our study). It was also worth noting that 1 patient developed dependence on corticosteroids after vaccination. Attacks occurred when the daily dosage was reduced or stopped (at least 1 drop per day).

**Table 3:** The comparison of medication between relapses before and after COVID-19 vaccination.

	**Before vaccination**	**After vaccination**	***P*-value**
Attack frequency (times/year)	3.56 ± 2.07	9.11 ± 7.34	**0.044** ^*^
Mean corticosteroids dosage (drops/day)	1.80 ± 1.69	2.25 ± 1.75	0.588
Mean GCV dosage (drops/day)	1.40 ± 1.90	2.67 ± 2.18	0.193
Mean IOP-lowering drug dosage (drops/day)	1.89 ± 2.76	3.00 ± 3.07	0.444
Corticosteroids dependence	0/12	1/11	0.500

GCV, ganciclovir; IOP, intraocular pressure. Statistically significant values were marked in bold with ^*^.

## 4. Discussion

In the current retrospective study, we reported 12 PSS patients suffering from relapses after any dose of inoculation of inactivated COVID-19 vaccines (Sinopharm, China) and reviewed their previous relapses simultaneously. Patients suffered from higher IOP, more KPs during the relapse following vaccination; higher dose of IOP-lowering drugs and corticosteroids were needed in both acute control and non-recurrent maintenance.

Ocular adverse events after inoculating COVID-19 vaccines were previously found in separate small cohorts worldwide, covering eyelid, cornea and ocular surface, retina, uvea, nerve and vessel (16, 17). It was usually hypothesized that ocular manifestations were induced *via* an immune response elicited by the vaccine (17). Cases of uveitis triggered or re-activated by SARS-CoV-2 have been described (18). The ocular manifestations of vaccination appeared to overlap with that reported in COVID-19 infection, suggesting a common pathway between virus and vaccine-mediated immune response in human. This could be explained by the innate and adaptive immune response primed by vaccines (19). Although the eye is considered a prototypic immune-privileged organ, the systemic derangement of immunity triggered by immunogenicity of COVID-19 vaccine may interfere with the stability of autoimmune diseases (20) and furthermore, induce uveitic relapses (as we hypothesized). Though the pathogenesis remained unclear, there had been several hypotheses, such as molecular mimicry, antigen-specific cell and antibody mediated hypersensitivity reactions, inflammatory damage induced by adjuvants, etc. (21). Currently, the pathogenesis of Posner-Schlossman syndrome is considered to be more viral infection than autoimmunity (22). However, the balance between cytomegalovirus (CMV) latency and opportunistic recurrences could be broken when the individual's systemic immunity is impaired, challenged or suppressed (systemic autoimmune diseases, the application of immunosuppressive agents or high dose of corticosteroids, etc.). Cytomegalovirus is an opportunistic pathogen which often establishes subclinical infection in immunocompetent individuals (23). Through evading immune surveillance and establishing latency, CMV could replicate in the iris, the ciliary body, cornea endothelium and the trabecular meshwork under the circumstance of immunosuppression (23, 24). Among the 12 participants in our study, the most frequent incentives found previously were tiredness, bad emotions and specific allergens, which may lead to temporary immune compromise. Despite no systemic autoimmune disease was diagnosed, it was quite difficult to conclude whether those patients were congenitally vulnerable to immune derangement or there existed the possibility that systemic or ocular immunogenicity contributed to PSS pathogenesis, apart from viral infections (since too many ambiguous variables were involved). Previously, the genetic polymorphism and upregulated circulating plasma protein levels of cytotoxic T-lymphocyte associated protein 4 (CTLA-4) and programmed cell death 1 (PD-1) were suggested relevant to PSS susceptibility in China (25). The concentrations of different immune mediators in the aqueous humor were also investigated linked with acute or chronic uveitis (15). In our study, unlike previous experience (no oral immunosuppressive agent was needed; the relapse could be controlled within 7.33 ± 6.51 days), in the relapse following COVID-19 vaccination, although only 1 patient received oral immunosuppressive agents for 3 months, the other 11 underwent a tough period controlling elevated IOP and uveitic rebounds (mean: 30.71 ± 34.74 days under higher regimen, poor responsiveness to corticosteroids). The dilemma of helping patients maintain quiet anterior chamber after COVID-19 vaccination may serve as evidence that the change of systemic immune stability may lead to PSS recurrence, though more studies in terms of mechanism remain needed. Because the anterior paracentesis was an invasive procedure, only those under high risk of viral infection [judged by clinical manifestations: obvious iris depigmentation or atrophic changes accompanied by coin-shaped and mutton-fat KPs on the nasal and superior nasal quadrants of corneal endothelium, etc. (26–28)] were recommended for aqueous humor sample test in our study. This also explained why we did not have control group; hardly could the negative predictive value be determined in this study. Although 3 patients were viral IgG positive, there is no direct evidence of the relationship between the specific IgG titer and acute viral replication in the aqueous humor. The IgG results acquired by enzyme-linked immunosorbent assay was considered to be less dependent on the timing of sampling and reflect historical infections (27, 29).

Currently, the literature of COVID-19 vaccines-related uveitic relapse was rare. Uveitic adverse events previously reported included new onset of posterior uveitis (30), relapse of Vogt-Koyanagi-Harada (VKH) disease and Bechet's disease, etc. (31). Different attention should be paid to relapses from new onsets of uveitis. New onset of PSS has not been observed worldwide so far; to our knowledge, this is the first study reporting the PSS relapse possibly triggered by inactivated COVID-19 vaccines. Previously, Pang et al. (32) reported a case series of ocular adverse events after inactivated vaccine in China. All the three patients in their study denied medical history or family history of autoimmune disease, so they speculated the enhancement of immune response might play an important role in these uveitis cases. Pichi et al. (9) reported some acute adverse events after inactivated COVID-19 vaccination also developed by Sinopharm. In their observation, the mean time of attack was 5.2 days (range: 1–10 days), which was close to ours (5.27 days, range 1–13). Jain et al. (33) reported a single case of bilateral uveitic relapse after COVID-19 vaccination (Covishield–Serum Institute of India). Since the COVID-19 vaccines work by upregulating both the humoral and cellular immunity, especially in the initial week post vaccination, they speculated the pathogenesis might be the uncontrolled activation of the innate immune system. The patient enrolled in their study had been diagnosed juvenile idiopathic arthritis before, so it was of great possibility that the immune derangement operated during the relapse. Similarly, the role of autoimmune regulation in Posner-Schlossman syndrome was also recognized in several studies (25, 34, 35). Till now, the pathogenesis of PSS is still unproven. Evidence has evolved infection, injury, and autoimmune drive can all contribute to hypertensive uveitis (1). VKH reactivation (36) and multiple evanescent white dot syndrome (37) after BNT162b2 mRNA vaccines were also reported worldwide. Unlike the BNT162b2 mRNA vaccine [comprised of nucleoside-modified mRNA, could translate into SARS-CoV-2 spike protein after entry and express on the surface of the host cells, which induces neutralizing antibody and cellular immune responses against it (38, 39)], inactivated vaccines depend on adjuvants to achieve robust antibody immune response (mainly humoral immune responses) (40, 41). Due to the influence of adjuvants on the adaptive and innate arms of the immune system, the regulation of autoimmune condition might contribute to latent viral reactivation and uveitic relapse. However, the precise pathogenesis frequently remains unclear. Potential problems of humoral and cellular immune responses and immunity of participants on a larger scale need clarification in order to further illustrate the dynamics of immune status after vaccination. Notably, complete resolution was achieved both in anterior uveitis after BNT162b2 (37) and ocular adverse events after inactivated vaccine (9). In our study, all the patients suffered from more attacks and harder uveitic control post-vaccination. This may also indicate the importance of focusing on the relapses of pre-existing hypertensive uveitis. Under-appreciated recurrence of PSS may expand the spectrum and accelerate disease progression, regardless of previous medication. Although the causal relationship could not be established from this study, it was suggested attention should also be paid to vulnerable uveitis population.

Our study had several limitations. It would be better to include serum immunological tests to evaluate systemic immune status of patients, which may also provide clues for mechanism exploration. Longer period of follow up was needed to assess the speed of uveitic progression and drug responsiveness. Viral DNA PCR test was not performed, otherwise a clearer hypothesis on the relationship between viral reactivation and vaccination might be proposed. However, we were acknowledged that mysteries still hided in the immune regulation of PSS relapses, viral opportunistic reactivation, as well as vaccination. Thus, we temporarily focused on reporting the phenomenon and the incidence rate of PSS relapse following inactivated COVID-19 vaccination in east China. In conclusion, clinical ophthalmologists should pay special attention to PSS vulnerability to inactivated COVID-19 vaccine doses. Patients should avoid inoculating any dose of inactivated COVID-19 vaccine during recurrence; even during the relapse-free period, patients should be fully informed of the risk of relapse and the possibility of difficulty in controlling acute attacks; frequent follow ups and timely adjustments of current medication regimen are recommended both before and after vaccination, suggested by our study.

## Data availability statement

The original contributions presented in the study are included in the article/supplementary material, further inquiries can be directed to the corresponding author.

## Ethics statement

The studies involving human participants were reviewed and approved by the Ethics Committee of EENT Hospital of Fudan University. The patients/participants provided their written informed consent to participate in this study.

## Author contributions

QS and YS: wrote the original draft, formal analysis, and conceptualization. RZ: conceptualization, reviewed and edited the manuscript, and supervision. XF and YY: validation, investigation, and data curation. XK: designed and guided the whole study. All authors contributed to the article and approved the submitted version.

## References

[1] Megaw R Agarwal PK. Posner-Schlossman syndrome. Surv Ophthalmol. (2017) 62:277–85. 10.1016/j.survophthal.2016.12.005 28012873

[2] Jap A Sivakumar M Chee SP. Is Posner Schlossman syndrome benign? Ophthalmology. (2001) 108:913–8. 10.1016/S0161-6420(01)00551-6 11320022

[3] Zhou F Yu T Du R Fan G Liu Y Liu Z. Clinical course and risk factors for mortality of adult inpatients with COVID-19 in Wuhan, China: a retrospective cohort study. Lancet. (2020) 395:1054–62. 10.1016/S0140-6736(20)30566-3 32171076 PMC7270627

[4] Ita K. Coronavirus Disease (COVID-19): current status and prospects for drug and vaccine development. Arch Med Res. (2021) 52:15–24. 10.1016/j.arcmed.2020.09.010 32950264 PMC7832760

[5] Ciotti M Ciccozzi M Pieri M Bernardini S. The COVID-19 pandemic: viral variants and vaccine efficacy. Crit Rev Clin Lab Sci. (2022) 59:66–75. 10.1080/10408363.2021.1979462 34598660

[6] Zhao J Zhao S Ou J Zhang J Lan W Guan W. COVID-19: Coronavirus vaccine development updates. Front Immunol. (2020) 11:602256. 10.3389/fimmu.2020.602256 33424848 PMC7785583

[7] Ng XL Betzler BK Testi I Ho SL Tien M Ngo WK. Ocular adverse events after COVID-19 vaccination. Ocul Immunol Inflamm. (2021) 29:1216–24. 10.1080/09273948.2021.1976221 34559576 PMC8477588

[8] ElSheikh RH Haseeb A Eleiwa TK Elhusseiny AM. Acute uveitis following COVID-19 vaccination. Ocul Immunol Inflamm. (2021) 29:1207–9. 10.1080/09273948.2021.1962917 34379565

[9] Pichi F Aljneibi S Neri P Hay S Dackiw C Ghazi NG. Association of ocular adverse events with inactivated COVID-19 vaccination in patients in Abu Dhabi. JAMA Ophthalmol. (2021) 139:1131–5. 10.1001/jamaophthalmol.2021.3477 34473209 PMC8414361

[10] Chau CYC Chow LLW Sridhar S Shih KC. Ophthalmological considerations for COVID-19 vaccination in patients with inflammatory eye diseases and autoimmune disorders. Ophthalmol Ther. (2021) 10:201–9. 10.1007/s40123-021-00338-1 33675508 PMC7936587

[11] Haseeb AA Solyman O Abushanab MM Abo Obaia AS Elhusseiny AM. Ocular complications following vaccination for COVID-19: a one-year retrospective. Vaccines. (2022) 10:342. 10.3390/vaccines10020342 35214800 PMC8875181

[12] Perez VL Caspi RR. Immune mechanisms in inflammatory and degenerative eye disease. Trends Immunol. (2015) 36:354–63. 10.1016/j.it.2015.04.003 25981967 PMC4563859

[13] Chan NS Chee SP Caspers L Bodaghi B. Clinical features of CMV-associated anterior uveitis. Ocul Immunol Inflamm. (2018) 26:107–15. 10.1080/09273948.2017.1394471 29172842

[14] Zhao J Chen W Huang X Peng S Zhu T Deng Z. Serum Th1 and Th17 related cytokines and autoantibodies in patients with Posner-Schlossman syndrome. PLoS ONE. (2017) 12:e0175519. 10.1371/journal.pone.0175519 28384257 PMC5383301

[15] Pohlmann D Schlickeiser S Metzner S Lenglinger M Winterhalter S Pleyer U. Different composition of intraocular immune mediators in Posner-Schlossman-syndrome and Fuchs' Uveitis. PLoS ONE. (2018) 13:e0199301. 10.1371/journal.pone.0199301 29944680 PMC6019249

[16] Lee YK Huang YH. Ocular manifestations after receiving COVID-19 vaccine: a systematic review. Vaccines. (2021) 2021:9. 10.3390/vaccines9121404 34960150 PMC8709261

[17] Ng XL Betzler BK Ng S Chee SP Rajamani L Singhal A. The eye of the storm: COVID-19 vaccination and the eye. Ophthalmol Ther. (2022) 11:81–100. 10.1007/s40123-021-00415-5 34914035 PMC8675299

[18] Sanjay S Mutalik D Gowda S Mahendradas P Kawali A Shetty R. Post coronavirus disease (COVID-19) reactivation of a quiescent unilateral anterior uveitis. SN Compr Clin Med. (2021) 3:1843–7. 10.1007/s42399-021-00985-2 34124585 PMC8184259

[19] Kang SM Compans RW. Host responses from innate to adaptive immunity after vaccination: molecular and cellular events. Mol Cells. (2009) 27:5–14. 10.1007/s10059-009-0015-1 19214429 PMC6280669

[20] Wraith DC Goldman M Lambert PH. Vaccination and autoimmune disease: what is the evidence? Lancet. (2003) 362:1659–66. 10.1016/S0140-6736(03)14802-7 14630450

[21] Cunningham ET Jr Moorthy RS Fraunfelder FW Zierhut M. Vaccine-associated uveitis. Ocul Immunol Inflamm. (2019) 27:517–20. 10.1080/09273948.2019.1626188 31247152

[22] Takusagawa HL Liu Y Wiggs JL. Infectious theories of Posner-schlossman syndrome. Int Ophthalmol Clin. (2011) 51:105–15. 10.1097/IIO.0b013e31822d6ab4 21897144

[23] La Distia Nora R Putera I Mayasari YD Hikmahwati W Pertiwi AM Ridwan AS. Clinical characteristics and treatment outcomes of cytomegalovirus anterior uveitis and endotheliitis: a systematic review and meta-analysis. Surv Ophthalmol. (2022) 67:1014–30. 10.1016/j.survophthal.2021.12.006 34954093

[24] Daicker B. Cytomegalovirus panuveitis with infection of corneo-trabecular endothelium in AIDS. Ophthalmologica. (1988) 197:169–75. 10.1159/000309939 2852787

[25] Huang X Liu X Ye Y Zhang T Mei S Zhu T. Polymorphisms and circulating plasma protein levels of immune checkpoints (CTLA-4 and PD-1) are associated with posner-schlossman syndrome in Southern Chinese. Front Immunol. (2021) 12:607966. 10.3389/fimmu.2021.607966 33717091 PMC7943469

[26] Sheng Q Zhai R Fan X Kong X. The analysis of dynamic changes and prognosis of Posner-Schlossman syndrome with cytomegalovirus infection and antiviral therapy. J Ophthalmol. (2021) 2021:6687929. 10.1155/2021/6687929 34123414 PMC8189808

[27] Sheng Q Zhai R Fan X Kong X. 2% Ganciclovir eye drops control Posner-Schlossman syndrome relapses with/without cytomegalovirus intraocular reactivation. Front Med. (2022) 9:848820. 10.3389/fmed.2022.848820 35355609 PMC8959537

[28] Kam KW Wong CH Ho M Sze RKH Chan PKS Young AL. Iris depigmentation in the prediction of cytomegalovirus anterior uveitis. Ocul Immunol Inflamm. (2021) 20:1–6. 10.1080/09273948.2021.1952277 34283680

[29] Lenglinger M Schick T Pohlmann D Pleyer U. Cytomegalovirus-positive posner-schlossman syndrome: impact on corneal endothelial cell loss and retinal nerve fiber layer thinning. Am J Ophthalmol. (2022) 237:290–8. 10.1016/j.ajo.2021.12.015 34998717

[30] Pan L Zhang Y Cui Y Wu X. Bilateral uveitis after inoculation with COVID-19 vaccine: a case report. Int J Infect Dis. (2021) 113:116–8. 10.1016/j.ijid.2021.09.075 34601147 PMC8482656

[31] Song H Zhao C Zhang M. There is no evidence that inactivated COVID-19 vaccines increase risks of uveitis flare. Vaccines. (2022) 10:1680. 10.3390/vaccines10101680 36298545 PMC9612251

[32] Pang K Pan L Guo H Wu X. Case report: associated ocular adverse reactions with inactivated COVID-19 vaccine in China. Front Med. (2022) 8:823346. 10.3389/fmed.2021.823346 35111790 PMC8801805

[33] Jain A Kalamkar C. COVID-19 vaccine-associated reactivation of uveitis. Indian J Ophthalmol. (2021) 69:2899–900. 10.4103/ijo.IJO_1435_21 34571678 PMC8597484

[34] Huang X Xu Y Chen W Zhu T He L Wang S. The genetic contribution of HLA-E^*^01:03 and HLA-E^*^01:03-G^*^01:01 to Posner-Schlossman syndrome in southern Chinese. Ann Transl Med. (2019) 7:749. 10.21037/atm.2019.11.70 32042765 PMC6989981

[35] Zhao J Huang XS Peng SM Mei SY Xu YP. A novel HLA-G allele, HLA-G^*^01:01:01:07, was identified in a Chinese patient with Posner-Schlossman syndrome. HLA. (2017) 90:136–40. 10.1111/tan.13058 28557312

[36] Papasavvas I Herbort CP Jr. Reactivation of Vogt-Koyanagi-Harada disease under control for more than 6 years, following anti-SARS-CoV-2 vaccination. J Ophthalmic Inflamm Infect. (2021) 11:21. 10.1186/s12348-021-00251-5 34224024 PMC8256412

[37] Rabinovitch T Ben-Arie-Weintrob Y Hareuveni-Blum T Shaer B Vishnevskia-Dai V Shulman S. Uveitis after the BNT162b2 mRNA vaccination against SARS-CoV-2 infection: a possible association. Retina. (2021) 41:2462–71. 10.1097/IAE.0000000000003277 34369440

[38] Lamb YN. BNT162b2 mRNA COVID-19 vaccine: first approval. Drugs. (2021) 81:495–501. 10.1007/s40265-021-01480-7 33683637 PMC7938284

[39] Sadarangani M Marchant A Kollmann TR. Immunological mechanisms of vaccine-induced protection against COVID-19 in humans. Nat Rev Immunol. (2021) 21:475–84. 10.1038/s41577-021-00578-z 34211186 PMC8246128

[40] Kumar A Meldgaard TS Bertholet S. Novel platforms for the development of a universal influenza vaccine. Front Immunol. (2018) 9:600. 10.3389/fimmu.2018.00600 29628926 PMC5877485

[41] Fathizadeh H Afshar S Masoudi MR Gholizadeh P Asgharzadeh M Ganbarov K. SARS-CoV-2 (Covid-19) vaccines structure, mechanisms and effectiveness: a review. Int J Biol Macromol. (2021) 188:740–50. 10.1016/j.ijbiomac.2021.08.076 34403674 PMC8364403

